# Providers and women’s perspectives on opportunities, challenges and recommendations to improve cervical cancer screening in women living with HIV at Mbarara Regional Referral Hospital: a qualitative study

**DOI:** 10.1186/s12905-024-03239-0

**Published:** 2024-07-08

**Authors:** Collins David Agaba, Alexcer Namuli, Brenda Ainomugisha, Leevan Tibaijuka, Mackline Ninsiima, Joseph Ngonzi, Cecilia Akatukwasa, Asiphas Owaraganise

**Affiliations:** 1 Department of Obstetrics and Gynecology, Mbarara University of Science and Technology, P.O. Box 1410, Mbarara, Uganda; 2 Department of Physiology, Mbarara University of Science and Technology, Mbarara, Uganda; 3 Department of Obstetrics and Gynecology, Mbarara Regional Referral Hospital, Mbarara, Uganda; 4 Department of Epidemiology, School of Public Health, Makerere College of Health Sciences, Kampala, Uganda; 5 Faculty of Medicine, Mbarara University of Science and Technology, Mbarara, Uganda; 6 Infectious Diseases Research Collaboration, Kampala, Uganda

**Keywords:** Improving, Cervical Cancer screening, Women living with HIV, Clinical care providers

## Abstract

**Background:**

Cervical cancer screening uptake remains low despite being a critical prevention method for adult women living with HIV(WLHIV). These women experience greater incidence and persistence of high-risk human papillomavirus (HPV) and severe outcomes, including cervical cancer comorbidity and death.

**Objective:**

We explored the opportunities, challenges, and recommendations of clinical care providers and WLHIV to improve cervical cancer screening uptake among WLHIV in Southwestern Uganda.

**Methods:**

In a cross-sectional qualitative study from January to June 2021 at Mbarara Regional Referral Hospital, we interviewed six key informant clinical care providers and held four focus group discussions with women living with HIV. Data was coded using Atlas ti software and analysed using thematic inductive analysis.

**Results:**

The participants identified several prevailing opportunities for cervical cancer screening, including skilled clinical care workers, public awareness for demand creation, optimized clinic flow, provider-led referrals, and peer-led information sharing that ease clinic navigation and shorten participant throughput. However, challenges occurred due to standalone services resulting in double queuing, longer clinic visit hours, missed chances for screening alongside unsupported lower health facilities leading to crowding at the referral hospital, and inadequate patient privacy measures leading to shame and stigma and the misconception that cervical cancer is incurable. Integrating HPV-DNA testing in HIV services was perceived with ambivalence; some participants worried about the quality of sample collection, while others valued the privacy it offered. Optimising self-collected DNA testing and sufficient counselling were recommended to improve cervical cancer screening uptake.

**Conclusion:**

Opportunities for cervical cancer screening included trained clinical care professionals, increased public awareness, improved clinic flow, provider referrals, and peer education. Challenges, such as unsupported lower-level health facilities, misconceptions, inadequate patient privacy, and uncertainty about integrating HPV-DNA screening into HIV services, were cited. Adequate counselling and self-sample collection were recommended to foster screening. Our findings may guide healthcare programs integrating cervical cancer screening into HIV clinics to reach the 70% World Health Organisation targets by 2030.

**Supplementary Information:**

The online version contains supplementary material available at 10.1186/s12905-024-03239-0.

## Background

Globally, in 2020, approximately 500,000 women contracted cervical cancer, and 340,000 died from it despite available preventive and treatment options such as human papillomavirus (HPV) vaccination and screening and treatment of pre-cancer or early-stage cancer [1, 2]. Women living with HIV (WLHIV) experience a greater incidence and persistence of high-risk HPV infection and severe outcomes, including cervical cancer and death, than their HIV-negative counterparts [3–5]. In sub-Saharan Africa, 24.9% of cervical cancers have been diagnosed in WLHIV, and approximately 20% of cases are attributable to HIV, compared to 1.1% around the globe [3]. In Uganda, cervical cancer remains the most common malignancy, with estimated annual diagnoses of nearly 6,400 women and the leading cause of death of about 4,300 women [4]. Evidence shows declining cervical cancer prevalence worldwide, except in sub-Saharan Africa [4], primarily due to limited access to vaccination, low screening uptake and treatment of precancerous cervical lesions [6] amidst a generalised HIV epidemic [7, 8].

The World Health Organisation (WHO) recommends screening at least 70% of eligible women between the ages of 35 and 45 using a high-performance test by 2030 as one of the cervical cancer elimination strategies [2, 9]. Mathematical models also show that screening for cervical cancer twice in a lifetime between the ages 30 and 45 years combined with HPV vaccination could eliminate cervical cancer among WLHIV by 2120 [9, 10]. Uganda recommends HPV testing as the primary cervical cancer screening method and visual inspection with acetic acid (VIA) where HPV testing is unavailable or Papanicolaou (Pap) smear in post-menopausal women— repeated every three years for WLHIV [11]. The WLHIV can enter cervical cancer screening programs through various healthcare entry points, mainly the HIV clinics, sexual and reproductive health clinics, outreach programs, and occasionally self-testing options where available. Despite WLHIV interfacing with clinicians at least twice a year and the presence of policy guidelines, cervical cancer screening rates of 30% nationally and 27% for western Uganda [12] remain far below the WHO 70% target. At another Ugandan urban HIV clinic, approximately 44% of eligible women had ever screened for cervical cancer, with only 16.1% having been screened in the previous year [13].

Screen-and-treat strategies have been used in cervical cancer screening programs to improve efficiency and accessibility, especially in low-resource settings [14]. However, few studies have triangulated the nuanced perspectives of clinical care providers and users to optimize cervical cancer screening among WLHIV. In Sub-Saharan Africa, symptom debut [15, 16], disease awareness [17], and being referred to screening [18] have been cited as factors that promote cervical cancer screening. On the contrary, unfavourable geographical location and terrain [19], fear of embarrassment [20], high costs [21], and limited provider knowledge and skill [22] were cited as challenges to screening uptake. Amidst the above opportunities and obstacles, screening rates among WLHIV continue to lag behind the WHO-recommended 70% cervical cancer screening targets. Therefore, we conducted a qualitative study exploring the perspectives of clinical care providers and women on opportunities, challenges, and recommendations to improve cervical cancer screening uptake among eligible WLHIV at a tertiary hospital in Southwestern Uganda.

## Methods

### Study design and duration

We conducted a cross-sectional qualitative study to explore the perspectives of clinical care providers and WLHIV on the determinants of cervical cancer screening and how to improve cervical cancer screening uptake among eligible WLHIV from January to June 2021.

### Study setting

The study was conducted in the two outpatient clinics, the HIV and cervical cancer screening clinic of the Mbarara Regional Referral Hospital-Mbarara University of Science and Technology Complex in Mbarara City. The hospital complex serves 13 districts of Southwestern Uganda, with approximately 6 million people [23]. One clinic, the immunosuppression (ISS) clinic, is the highest-volume HIV clinic in the region, providing comprehensive HIV care to about 17,212 WLHIV as of September 2019 [24]. The HIV-related clinical activities included HIV testing and counselling and antiretroviral therapy. The other was the cervical colpopathology (CCP) clinic, offering cervical cancer screening to approximately 15 women daily [23]. The two clinics are open Monday through Friday, and any of the about 168 WLHIV seen in the ISS clinic daily could walk in or be referred by health workers to screen for cervical cancer in the CCP clinic two blocks away [24]. Also, WLHIV come into CCP from sexual health clinics, outreach programs, referrals from peripheral lower health centers, and occasionally self-testing options where available. The cervical cancer screening-related services include HPV-DNA testing, visual inspection with acetic acid (VIA), which is the commonest screening method, colposcopy, cervical biopsy, and Pap smear. The treatment options for precancerous cervical lesions include ablative therapy with thermocoagulation or cryotherapy and Loop Electrosurgical Excision Procedure (LEEP). At the CCP clinic, screening for cervical cancer involves educational counselling, HPV-DNA testing, followed by a triage of positive HPV cases using VIA. Some women access VIA screening and Pap smear testing without HPV testing. Colposcopy and cervical biopsy collection are also offered accordingly. Precancer cervical lesions based on VIA, Pap smear or biopsy are treated in the same clinic, while diagnosed cancer cases are referred to gynaecological and oncology departments of the same referral hospital for further management.

### Study participants and eligibility criteria

We studied adult WLHIV and their clinical care providers. We included WLHIV aged 24–49 years (to match the age range recommended for the VIA screening method) who were in care at the ISS clinic for at least one year before March 30, 2020, when Uganda announced the first COVID-19 lockdown. We excluded WLHIV who reported prior uterine removal or cervical cancer screening in the past year without medical record or who declined study participation. We included adult male and female clinical care providers who had delivered clinical services for at least three months at the ISS or CCP clinic.

### Sample size and sampling procedures

All participants were purposively sampled [25] based on the duration of service for clinical care providers and age, time of known HIV diagnosis, and previous cervical cancer screening for WLHIV. We conducted six (*n* = 6) key-informant interviews (KIIs) with clinical care providers selected from a list of HIV medical officers in the ISS clinic (*n* = 3) and all nurses/midwives and specialists in the CCP clinic (*n* = 3). We conducted four (*N* = 4) face-to-face focus group discussions (FGDs) with WLHIV balanced between those screened on schedule (2 FGDs) and those overdue for cervical cancer screening (2 FGDs) per the self-reported most recent screening date verified from medical records.

### Data collection procedures

A gender-balanced team of four (4) researchers (CDA, AO, CA, AN) trained in qualitative research methods conducted face-to-face semi-structured KIIs and FGDs using interview guides. The researchers explored topical domains, including general experiences in delivering or receiving HIV care and cervical cancer screening and the role of clinical care providers, WLHIV, and healthcare facilities in improving cervical cancer screening services. The opportunities for cervical cancer screening referred to ways WLHIV could access and undergo screening for cervical cancer. All data collection procedures occurred at the MRRH premises, workplace and clinic. We held all FGDs in a private waiting room in the CCP clinic after all women living with HIV due for cervical cancer screening had completed the cervical cancer screening exercise. Clinical providers’ KIIs were held in their routine consultation offices at the hospital. Only the study participants and researchers were present during the FGD or KII.

The semi-structured KII guide sought a general description of a typical day providing services at the CPP or ISS and any challenges or rewards at work. We then sought their views on what service users and clinical care providers could stop or continue doing to achieve cervical cancer screening targets in women living with HIV. Finally, clinical care providers reflected on whether there was anything else they felt was important to share with the study team on cervical cancer screening.

The FGD semi-structured interview guide explored broad descriptions of the day-to-day clinical services they receive at CCP and ISS clinics. We also sought their opinions on cervical cancer screening processes that seemed to work well or needed improvement, thereby allowing other unanticipated domains to be examined. To ensure cultural nuance, participants preferred the local language (Runyankole); fluent researchers conducted the FGDs.

Audio recordings were used to collect data from KIIs and FGDs. FGDs averaged 1.5 h, while the KIIs lasted about 50 min. Verbatim transcription of the field notes and audio recordings was immediately done following each KII or FGD. The research team transcribed audio recordings into English, and no KII or FGD was repeated.

### Data analysis

Our research team, consisting of four members (AO, CA, AN, CDA), developed an initial coding framework [26, 27]. We based it on the data we collected through interviews. We refined the framework deductively to align with the key thematic areas identified in the interview guides. We reviewed and refined the framework throughout the coding process to ensure accuracy and consistency. To ensure coding consistency, the four researchers collaboratively discussed and reached a consensus on how to code discrepant data segments. After constructing and reviewing salient themes, we summarized the main findings. Data were coded in Atlas.ti software. The consolidated criteria for reporting qualitative research (COREQ) checklist (supplementary file) for interviews and focus groups guided our study reporting [28].

### Ethical approvals

Ethical approval to conduct this study was obtained from the Mbarara University Research Ethics Committee (Protocol reference number 14/01–19). Written informed consent was obtained from all study participants. Only individuals over 18 years of age participated in this study.

## Results

The median age of the 27 women living with HIV was 38 years (range 25–49), and their median duration of known HIV diagnosis was seven completed years (range 2–26). The median age of six clinical care providers was 41 years; two were male, and their duration of clinical practice ranged from 4 to 23 years, as shown in Table 1.

**Table 1:** Baseline characteristics of study participants, *N* = 33, Mbarara Regional Referral Hospital, 2021

Characteristic	Clinical Care Providers(*n* = 6)	Women Living with HIV (*n* = 27)
Male	2	Not applicable
Female	4	27
Median age in years (range)	42(27–48)	38(25–49)
Years of clinical practice, range	3–23	Not applicable
Duration of known HIV diagnosis in years, range	Not applicable	2–13

Our analysis identified the perspectives of women living with HIV and their clinical care providers, which we summarised under three domains as challenges, opportunities, and recommendations to improve cervical cancer screening for eligible women at Mbarara Regional Referral Hospital. The detailed codebook is attached as a Supplemental Table 1.

### Opportunities for cervical cancer screening uptake among eligible WLHIV

#### Peer-led information sharing encourages cervical cancer screening

Participants reported learning about cervical cancer screening from peers, particularly those with personal experience. Two female participants living with HIV shared their comments:

“I have always heard about it, but I had never been instructed to go for screening, which is why I thank my friend who told me of the opportunity to be among those to be screened.” Women living with HIV, FGD.

“While the service cannot be provided at that ISS [HIV clinic] because it requires women’s privacy, we encourage those who screened to spread information and encourage their colleagues to come.” Provider KII.

“I have lived in Mbarara for thirty years. I started coming for treatment up there in 2011, but I had learned today that these services are available when [name] told me to come along and screen.” Women living with HIV, FGD.

#### Optimised clinic flow and provider-led referrals foster cervical cancer screening

Opportunities to enhance cervical cancer screening for women living with HIV were identified, including streamlining clinic flow and improving communication between clinical care providers and patients. At the clinic level, we noted opportunities in the client flow that would encourage WLHIV to screen for cervical cancer. Streamlined clinic flow and linkage support by peers, especially for women referred from lower-level health facilities or in-reach referrals from other hospital departments, was critical. Clear communication between clinical care providers and women during the screening process and prompts for cervical cancer screening for those due was also cited as crucial for improved service uptake.

“Those are not health workers [clinicians]; they are peer mothers [lay health workers]. And it is true that they escort us here and hand over files to nurses.” Women living with HIV, FGD.

“The patient flow in the cervical cancer clinic is haphazard. Some patients come voluntarily to the clinic to specifically screen for cervical cervix. In contrast, others are referred from lower health facilities or departments like OPD, especially when presenting symptoms. The health workers, therefore, must handle them depending on how they present themselves at the clinic.” Provider KII.

“Some are referred from lower health centres and seek cervical cancer screening because they know what they want. Others come to the usual outpatient department, and the clinician there advises them to undergo cervical cancer screening. Others get information from colleagues and seek cervical cancer screening.” Provider KII.

#### Public awareness about cervical cancer screening services improves uptake

Women living with HIV who were screened on schedule were generally reported to have received information about screening services via radio. This was especially true for those living in remote areas with limited access to information. The general public was increasingly informed about cervical cancer through local media, including radio and television, as well as educational outreach programs by the host health facility. As a result, awareness among the general population increased, leading to a higher demand for cervical cancer screening.

“Some people trying to advertise their herbal medicine on TV also teach a few things about cervical cancer and the need for screening.” Women living with HIV, FGD.

“To get to know that I needed to go for screening, I heard on the radio that there was free [cervical cancer] screening at [name]’s hospital.” Women living with HIV, FGD.

Clinical care providers equally suggested that the hospital leverage a combination of referral systems and community engagement via radio announcements and education to reach women for cervical cancer screening.

“This regional referral hospital deals with many patients from different districts; some come from nearby health centres as referrals, and others come from the community. The majority come from the community because they get announcements from the radio, or sometimes during community work, we go and educate them.” Provider KII.

#### Available skilled clinical care workers to perform cervical cancer screening

The participants self-perceived adequate and continuous training in cervical cancer screening and management, allowing them to conduct multiple procedures related to cervical cancer. The clinical care providers reported participating in exercises in cervical cancer screening and management of women based on the screening outcomes. They did not stop at initial training but had continuously updated skills depending on how the screening methods and guidelines evolved. Having received training in cervical cancer screening, a clinical care provider reports being proficient in several screening and precancerous cervical lesions treatment procedures. Clinical care workers acknowledge that continuous training has familiarised them with crucial cervical cancer screening and management techniques.

“From the beginning, we were trained to do VIA, whic is Visual Inspection with Acetic acid. Along the way, we added on doing pap smears, looking at colposcopy, and treating pre-cancers with cryotherapy or thermos-coagulation. We are also trained in assisting doctors doing LEEP, so we added something every other time.” Provider KII.

“After the HPV DNA testing guidelines were realized, I attended the trainer of trainees workshop organized by MoH [Ministry of Health]. As soon as I returned, I trained my colleagues during CMEs [continuous medical education] my colleagues.” Provider KII.

### Challenges in cervical cancer screening uptake among eligible WLHIV

#### Fragmentation of services leads to missed opportunities for screening

The CPP clinic for cervical cancer screening is held parallel to the ISS HIV clinic, causing difficulties for women attending both due to high client loads. Women expressed concerns about arranging the two service points: cervical cancer screening in the CPP clinic away from the ISS HIV clinic. There were implications; for example, a woman living with HIV found attending two clinics on the same day challenging. Worse still, each clinic had high client loads, and screening supplies were perceived to be intermittently dependent on the implementing partner financing them.

“However much they tell us how cancer may kill us before even the HIV does, they don’t understand moving from one long queue for getting the drugs to another long one for screening, which disturbs us much.” Women living with HIV, FGD.

“It is done, and at one point, we used to tell them that if their file does not have the stamp confirming they have been screened, then they would not get the drugs, but the ladies would even cry, saying they have to go and attend to their businesses and we are holding them back for the whole day. The fact that they have to endure the queue for screening and then the one for getting the drugs somehow disturbs these ladies.” Provider, KII.

“Often cervical cancer screening is intermittent and usually parallel depending on the implementing partner or funding project in the surrounding communities. HPV-DNA kits were expensive and supplied by the clinic; once the project supporting integration ended, so did the screening.” Women living with HIV, FGD.

#### Misconceiving cervical cancer as a “death sentence” hinders women from getting screened

Most women living with HIV reported that they generally perceived cancer diagnosis as a death sentence. Due to the high number of deaths associated with different cancers, being diagnosed with cervical cancer is considered to herald death. Some participants said they would prefer to remain ignorant of their cervical cancer status rather than learn about it and suffer mentally—described as “what I don’t know doesn’t kill me.”

“Others may not want to listen because they think what comes next after informing me that they have cancer is death, so they prefer to live in ignorance about it; counselling them not to fear may help.” Provider KII.

“I feared so much at first, but the last two, I was not afraid; I learned it is a check-up like other medical checks.” Women living with HIV, FGD.

#### Privacy concerns hinder women’s uptake of cervical cancer screening

Available standard cervical cancer screening options, such as VIA, Pap smear or collecting cervical biopsy, require women to undress and have a speculum placed into the vaginal canal to visualise the cervix. However, women feel embarrassed about being naked, which makes individuals reluctant to screen for cervical cancer.

“Some women are shy and fear being seen completely naked by the -doctor, not knowing that when the disease knocks them down, it will still be the same doctor to treat them; putting a screen is very fine.” Women living with HIV, FGD.

“As you can imagine, most women feel embarrassed during the cervical inspection. The self-collected HPV-DNA samples are, in a way, addressing this gap.” Provider, KII.

“They always say that they are not prepared to have the screening, given that the screening rotates around their privacy.” Provider, KII.

#### Lack of support to lower health facilities hinders cervical cancer screening

The participants perceived that lower health facilities lacked adequate capacity to implement cervical cancer screening. They felt that there was insufficient government support for essential supplies and tools to implement cervical cancer screening. Few clinical care providers had trained to screen and treat cases of cervical lesions adequately. At the same time, some health workers had received training of limited scope, mainly focused on screening and missed treatment and management, especially the early pre-cancerous stages. Others had transferred to other facilities.

“The major problem is few health workers being trained about cervical cancer screening. They may have the information but not know about the procedure; they need both to become fully competent.” Provider KII.

“Cervical cancer screening within the HIV clinic depends on the prevailing situation. Guidelines on cervical cancer screening are available at district hospitals and level-IV health centres, but they don’t routinely carry out screening.” Provider, KII.

“By the way, my first screening was at the health centre, although when I returned for the next one, they said the nurse no longer works there.” Women living with HIV, FGD.

### Recommendations to improve cervical cancer screening uptake among eligible women living with HIV

#### Adequately counselling women about cervical cancer screening

While clinical care providers knew what cervical cancer screening entails, women recommended improving pre-screening counselling. The clinical team was not meeting the expectations of women regarding the screening process and the potential outcomes. Clients anticipated peer-led counselling similar to that in the HIV clinics where peers living with HIV provide ongoing psychosocial counselling and support to newly diagnosed ones.

“When we come, we find the screen on and join others to watch the nurse explaining how one undergoes screening; when the nurse calls you in, she assumes you have prior knowledge of the screening process.” Women living with HIV, FGD.

“The other day, they taught about collecting samples, but I thought that was for those with previous experience; they should do counselling just like our peers in our clinic.” Women living with HIV, FGD.

#### Optimising clinic flow for self-sample collection improves screening method choice

We noted that WLHIV and their clinical care providers considered access to more cervical cancer screening options to choose from, such as up-scaling self-sampled HPV-DNA testing, to be an opportunity to improve cervical cancer screening and management of positive lesions. Women perceived self-collected HPV screening as less embarrassing and painful than VIA, and most felt they understood how to take a self-sample upon instruction. However, both clinical providers and women expressed heterogeneous views on the efficacy of self-sampling approaches. Most women living with HIV were unfamiliar with self-sampling, albeit voiced optimism. Mainly, the clinical care provider from the HIV clinic was skeptical about the capability of women living with HIV to collect their HPV-DNA samples.

“I am hopeful that most of the current challenges will reduce once we fully switch to HPV-DNA testing…; women can just be guided on sample collection, where to deposit it, and where to get her results.’’ Provider, KII.

“Unless the midwives help these women to collect those DNA samples, our common women are only familiar with urine samples; will they collect good samples sincerely?” Provider, KII.

“On that new one [HPV-DNA], most of us were hearing about it for the first time, but as a nurse helped me to collect my sample, it was not as painful or shaming as the usual one [VIA], she said the next one I should collect on my own, and the process seemed easy so that I will try,” Women living with HIV, FGD.

“With that rechargeable battery thermocoagulation and portable colposcope, I think we can increase outreach activities and even train more health center midwives.” Provider KII.

## Discussion

Cervical cancer is a significant health concern for women in HIV-burdened SSA, where screening coverage is low, impacting families and society [29]. In this qualitative study, providers and clients generated ideas on opportunities, challenges, and recommendations to improve cervical cancer screening uptake among eligible women living with HIV. The participants identified various opportunities for cervical cancer screening, such as having skilled clinical care workers, raising public awareness, optimising clinic flow, having provider-led referrals, and sharing peer-led information. However, screening for cervical cancer was challenging due to fragmented services, lack of support for lower health facilities, inadequate patient privacy measures, and the misconception that screening heralds cancer—a “death sentence”. Despite recommendations for adequate counselling and self-collected DNA testing [30], WLHIV and clinical care providers held contradictory perceptions regarding offering both cervical cancer screening and HIV services at the same location during a single visit. Our analysis gathered perspectives from providers and women living with HIV, complementing existing literature [31, 32]. Rather than focusing on barriers and facilitators, we sought participants’ insights on the entire cervical cancer screening process, providing a comprehensive range of perspectives on improving it.

In this study, women and clinical care providers suggested that self-collected HPV-DNA testing could potentially address privacy concerns with other screening methods. This is similar to what Joseph et al. in Uganda [33] and Obiri-Yeboah et al. in Ghana [34] found, that the majority of women felt capable of learning the self-sample collection procedure, which they believed would reduce embarrassment, shame, and pain associated with the commonly available VIA method. However, it was surprising that women living with HIV and clinical care providers were ambivalent about self-collected HPV testing and integrating cervical cancer screening in HIV clinics. Screening programs must address women’s anticipated delays and double queuing at HIV clinics and cervical cancer screening venues such as as described by Ninsiima and colleagues [24]. Additionally, in-service educational programs specifically designed for HIV care and treatment clinicians can address knowledge gaps among healthcare workers regarding HPV self-collection. These programs can disseminate information on the efficacy of self-collected samples and explain the benefits for women, such as increased access, convenience, and privacy [35].

Our findings regarding the lack of support for lower-level health facilities align with existing literature from sub-Saharan Africa. Studies point to similar bottlenecks in the lower level health facilities, such as staff shortages and high turnover, which ultimately hinder the implementation of the cervical cancer “screen-and-treat” strategy [36, 37]. Leveraging community health structures such as community health volunteers for mobilisation can expand screening and reach lower-level facilities to meet community screening demand. Private and non-governmental partners can also address public clinical care system gaps, like personnel and supply shortages identified in this study, to improve cervical cancer screening.

This study emphasises the importance of providing targeted training to clinical care providers and counselling eligible women to optimise optimize the integration of HPV-DNA testing into HIV clinic workflows. This is due to uncertainties surrounding self-collected HPV testing, concerns about self-efficacy in self-sampling [32], and results delivery [29] reported elsewhere. Additionally, low uptake of self-collected HPV testing was registered in Kenya among 25-34-year-old women at multi-disease community health fairs [38], highlighting the importance of tailoring HPV-based self-collection strategies to address the specific needs of women at different age groups. Beneficiary-collected samples being less accurate than clinician-collected ones due to potential user error and lack of familiarity with the procedure have been reported [39]. Giving clear instructions with illustrations and reassuring women can boost self-efficacy and help achieve the 70% screening target.

Streamlined clinic flow, optimised provider referral for testing and amplified community awareness via mass media were cited as opportunities to improve cervical cancer screening. Similar studies show that creating community awareness [40, 41] and enhancing provider-initiated referrals [13, 42] could improve overall cervical cancer screening uptake. This implies that standalone strategies might not improve screening uptake. Moreover, evidence shows that cervical cancer screening uptake was consistently lower than awareness levels in Kenya at 45% versus 84% [43], Uganda at 44% versus 98% [13], and Botswana at 100% vs. 24% [16], respectively. Our study underscores the need to move beyond awareness-only campaigns. Efforts to improve clinic efficiency, strengthen referral processes, and leverage mass media for targeted outreach can be crucial in translating awareness into action and ultimately expanding screening access.

Our study strength included triangulating data from the perspectives of clinical care teams who provide cervical cancer screening services and WLHIV who are service users. However, we also had some limitations. We did not differentiate quotes from specialists, nurses, and midwives to ensure anonymity in a small clinical staff unit. Our findings may not be generalizable to other populations due to our focus on specific HIV and cervical cancer clinics within a single regional referral hospital. However, the hospital serves a broader range of patients from neighbouring areas and regions with high HIV prevalence. We also enrolled WLHIV aged 25–49 years to match the VIA screening method precondition, limiting generalizability to younger and older women living with HIV. Finally, COVID-19 and its response measures may have influenced the study and cervical cancer screening activities as the number of screened women declined during the pandemic.

## Conclusions

This research explored the perspectives of women and clinical care providers on opportunities, challenges, and recommendations to enhance cervical cancer screening for the priority population of women living with HIV in Southwestern Uganda. Continuous and expanded efforts to deliver screening methods at user-centric and flexible venues for women living with HIV, alongside increasing community awareness and addressing integration concerns into HIV clinics, are crucial. Healthcare managers and programs in similar settings could use our findings to improve cervical cancer screening in women with HIV. Further research is needed to understand how expanded cervical cancer screening options integrated within HIV service points can be optimised to achieve the WHO cervical cancer screening targets by 2030.

### Electronic supplementary material

Below is the link to the electronic supplementary material.

Supplementary Material 1

Supplementary Material 2

## Data Availability

The supplementary material contains the codebook, and data are available upon reasonable request to the Faculty of Medicine at Mbarara University of Science and Technology through the corresponding author.

## Abbreviations

CCP: Cervical colpopathology
COREQ: Consolidated criteria for reporting qualitative research
DNA: Deoxyribonucleic acid
FGD: Focus group discussion
HPV: human papilloma virus
ISS: Immunosuppression
KII: Key informant interview
WHO: World Health Organisation
WLHIV: Women living with HIV
