# Supplementary material for: Providers and women’s perspectives on opportunities, challenges and recommendations to improve cervical cancer screening in women living with HIV at Mbarara Regional Referral Hospital: a qualitative study
Source: BMC Womens Health. 2024 Jul 8;24:392. doi: 10.1186/s12905-024-03239-0 (PMC11229203; doi:10.1186/s12905-024-03239-0)
Supplement: Supplementary file 1 — Supplementary Material 1 [file 12905_2024_3239_MOESM1_ESM.docx]

**Supplemental Table: Domains and themes from FGDs with women living with HIV and provider KIIs at MRRH, 2021.**

| **Domain** | **Theme** | **Women Living with HIV (FGDs)** | **Clinical care providers (KIIs)** |
| --- | --- | --- | --- |
| **Prevailing opportunities for cervical cancer screening among eligible Women Living with HIV** | Peer-led information sharing on cervical cancer screening. | “I have always heard about it, but I had never been instructed to go for screening, and that is why I thank my friend who told me of the opportunity to be among those to be screened” (Woman Living with HIV, FGD).  “I have lived in Mbarara for thirty years. I started coming for treatment up there in 2011, but I had gotten to know today that these services are available when [name] told me to come along and screen” (Woman Living with HIV, FGD). | “While the service cannot be provided at that ISS [HIV clinic] because it requires women's privacy, we encourage those who screened to spread information and encourage their colleagues to come” Provider KII |
|  | Optimized clinic flow and provider-led referrals for cervical cancer screening | “Those are not health workers [clinicians]; they are peer mothers [lay health workers]. And it is true that they escort us here and hand over our files to nurses”, Women living with HIV, FGD | “The patient flow in the cervical cancer clinic is haphazard. Some patients come voluntarily to the clinic to specifically screen for a cervix. In contrast, others are referred from lower health facilities or departments like OPD, especially when presenting symptoms. The health workers, therefore, must handle them depending on how they present themselves at the clinic” (Provider KII).  “Some are referred from lower health centres and seek cervical cancer screening because they know what they want. Others come to the usual outpatient department, and the clinician there advises them to undergo cervical cancer screening. Others get information from colleagues and seek cervical cancer screening” (Provider KII). |
|  | Public awareness for cervical cancer screeching services. | “Some of the people trying to advertise their herbal medicine on TV also teach a few things about cervical cancer and the need for screening” (Woman Living with HIV, FGD)  “To get to know that I needed to go for screening, I heard on the radio that there was free [cervical cancer] screening at [name]’s hospital” [Woman Living with HIV, FGD] | “This regional referral hospital deals with many patients from different districts; some come from nearby health centres as referrals, and others come from the community. The majority come from the community because they get announcements from the radio or sometimes during community work we go and educate them”, (Provider KII). |
|  | Optimising clinic flow and provider-led referrals for cervical cancer screening | “The patient flow in the cervical cancer clinic is haphazard. Some patients come voluntarily to the clinic to specifically screen for the cervical cervix. In contrast, others are referred from lower health facilities or departments like OPD, especially when presenting symptoms. The health workers, therefore, handle them depending on how they present themselves at the clinic” [KII participant]  “Some are referred from lower health centres and seek cervical cancer screening because they know what they want. Others come to the usual outpatient department, and the clinician there advises them to undergo cervical cancer screening. Others get information from colleagues and come looking for cervical cancer screening [KII participant] | “Those are not health workers [clinicians]; they are peer mothers [lay health workers]. And it is true that they escort us here and hand over files to nurses”, Women living with HIV, FGD |
|  | Skilled healthcare workers to perform cervical cancer screening |  | “From the beginning, we were trained to do VIA, that is, Visual Inspection with Acetic acid. Along the way, we added on doing pap smears and looking at colposcopy, treating pre-cancers with cryotherapy or thermos-coagulation. We are also trained in assisting doctors doing LEEP, so we add something every other time” (Provider KII).    “After the HPV DNA testing guidelines were realized, I attended the trainer of trainees workshop organized by MoH [Ministry of Health]. As soon as I returned, I trained my colleagues during CMEs [continuous medical education] my colleagues.” Provider KII. |
| **Prevailing challenges in cervical cancer screening among eligible women living with HIV** | Fragmentation of services leads to missed opportunities for screening. | “However much they tell us how cancer may kill us before even the HIV does, they don’t understand moving from one long queue for getting the drugs to another long one for screening which disturbs us much” (Woman Living with HIV, FGD).  “Often cervical cancer screening is intermittent and usually parallel depending on the implementing partner or funding project in the surrounding communities. HPV-DNA kits were expensive and supplied by the clinic; once the project supporting integration ended, so did the screening” (Woman Living with HIV, FGD). | “It is done, and at one point, we used to tell them that if their file does not have the stamp confirming they have been screened, then they would not get the drugs, but the ladies would even cry, saying they have to go and attend to their businesses and we are holding them back for the whole day. The fact that they have to endure the queue for screening and then the one for getting the drugs somehow disturbs these ladies” (Provider, KII). |
|  | Conceiving cervical cancer as a death sentence. | “I feared so much at first, but the last two, I was not afraid, I learned it is a check-up like other medical checks” (Woman Living with HIV, FGD). | “Others may not want to listen because they think what comes next after informing me that they have cancer is death, so they prefer to live in ignorance about it; counselling them not to fear may help” (Provider, KII). |
|  | Current screening options elude the privacy of patients. | “Some women are shy and fear being seen completely naked by the -doctor, not knowing that when the disease knocks them down, it will still be the same doctor to treat them; putting a screen is very fine” (Woman Living with HIV, FGD). | “As you can imagine, most women feel embarrassed during the cervical inspection. The self-collected HPV-DNA samples are in a way addressing this gap” (Provider, KII)  “They always say that they are not prepared to have the screening, given that the screening rotates around their privacy” (Provider, KII). |
|  | Lower health facilities are not well-supported to conduct the screening. | “By the way, my first-time screening was at the health centre, although when I returned for the next one, they said the nurse no longer works there” (Woman Living with HIV, FGD). | “The major problem is few health workers being trained about cervical cancer screening. They may have the information but not know about the procedure; they need both to become competent” (Provider KII).  “Cervical cancer screening within the HIV clinic depends on the prevailing situation. Guidelines on cervical cancer screening are available at district hospitals and level-IV health centres, but they don’t routinely carry out screening” (Provider, KII). |
| **Recommendations to improve cervical cancer screening in eligible Women Living with HIV.** | Adequately counsel women about cervical cancer screening. | “When we come, we find the screen on and join others to watch the nurse explaining how one undergoes screening; when the nurse calls you in, she assumes you have prior knowledge of the screening process” (Women Living with HIV, FGD).  “The other day, they taught about collecting samples, but I thought that was for those with previous experience; they should do counselling just like our peers in our clinic” (Women Living with HIV, FGD) |  |
|  | Optimize self-sampling techniques | “On that new one [HPV-DNA], most of us were hearing about it for the first time, but as a nurse helped me to collect my sample, it was not as painful or shaming as the usual one [VIA], she said the next one I should collect on my own, and the process seemed easy so that I will try,” (Women living with HIV, FGD). | “I am hopeful that most of the current challenges will reduce once we fully switch to HPV-DNA testing…; women can just be guided on sample collection, where to deposit it, and where to get her results’’ (Provider, KII).  “Unless the midwives help these women to collect those DNA samples, our common women are only familiar with urine samples; will they collect good samples sincerely?” (Provider, KII).  “With that rechargeable battery thermocoagulation and portable colposcope, I think we can increase outreach activities and even train more health centre midwives” (Provider KII). |

HPV= Human papillomavirus, MRRH= Mbarara Regional Referral Hospital
