# Supplementary material for: Providers and women’s perspectives on opportunities, challenges and recommendations to improve cervical cancer screening in women living with HIV at Mbarara Regional Referral Hospital: a qualitative study
Source: BMC Womens Health. 2024 Jul 8;24:392. doi: 10.1186/s12905-024-03239-0 (PMC11229203; doi:10.1186/s12905-024-03239-0)
Supplement: Supplementary file 2 — Supplementary Material 2 [file 12905_2024_3239_MOESM2_ESM.docx]

**Consolidated criteria for reporting qualitative research (COREQ): a 32-item checklist**

Adapted from Tong, Sainsbury, & Craig, 2007[1]

**Domain 1: Interview Context**

1. Interviewers: CDA, AO, CA, AN (line 136).
2. Credentials: AN (BSN), CDA, AO, BA & LT (MDs), CA (MPH), MN (MSc), JN (PhD).
3. Roles: Clinicians, academics, social scientist.
4. Gender: Balanced (male: CDA & AO, female: AN & CA).
5. Training: CA & AO led data collection (experienced in qualitative research with WLHIV)
6. Relationship: Pre-existing professional relationships as clinicians of WLHIV
7. Participant information: The interview guide explained the purpose, consent, and procedures.
8. Mitigating bias: Being clinicians, we relied on our training, interview guides, and external non-clinician (CA) leading data collection to minimize bias.

**Domain 2: Interview Selection**

1. Approach: Combined deductive and inductive approach for thematic analysis (lines 163-166)
2. Sampling: Purposive (line 127)
3. Method: Face-to-face (line 137)
4. Sample size: N=33 (WLHIV: 27, Clinicians: 6) (Table 1)
5. Non-participation: Two women declined participation over time constraints, not shown
6. Setting: Hospital (line 142)
7. Observers: Only participants and researchers (line 146)
8. Sample description: Sex, age, HIV diagnosis duration, years in service (Table 1)
9. Interview guide: Developed and pilot-tested
10. Repeat interviews: Not done
11. Recording: Audio (line 158)
12. Field notes: Yes (line 159)
13. Duration: KIIs: 50 min, FGDs: 1.5 hours (lines 158-159)
14. Data saturation: Not discussed
15. Transcript return: Not done

**Domain 3: Data Analysis**

1. Coders: Four (AO, CA, AN, CDA) (line 163)
2. Coding tree: Yes (we provided a supplementary coding table)
3. Theme derivation: Combined deductive & inductive approaches (lines 163-166)
4. Software: Atlas.ti (line 169)
5. Participant checking: Not done
6. Quotes: Yes (anonymized, illustrate key themes)
7. Data-finding consistency: Systematic approach, quotes support findings
8. Major theme clarity: Descriptive labels, quotes, interpretations
9. Minor theme clarity: Included screened & overdue participants

1. Tong A, Sainsbury P, Craig J: **Consolidated criteria for reporting qualitative research (COREQ): a 32-item checklist for interviews and focus groups**. *International journal for quality in health care* 2007, **19**(6):349-357.
